# HDAC8: A Promising Therapeutic Target for Acute Myeloid Leukemia

**DOI:** 10.3389/fcell.2020.00844

**Published:** 2020-09-04

**Authors:** Marco Spreafico, Alicja M. Gruszka, Debora Valli, Mara Mazzola, Gianluca Deflorian, Arianna Quintè, Maria Grazia Totaro, Cristina Battaglia, Myriam Alcalay, Anna Marozzi, Anna Pistocchi

**Affiliations:** ^1^Dipartimento di Biotecnologie Mediche e Medicina Traslazionale, Università degli Studi di Milano, Milan, Italy; ^2^Dipartimento di Oncologia Sperimentale, Istituto Europeo di Oncologia IRCCS, Milan, Italy; ^3^Cogentech, Società Benefit, Milan, Italy; ^4^Dipartimento di Oncologia ed Emato-Oncologia, Università degli Studi di Milano, Milan, Italy

**Keywords:** HDAC8, AML, PCI-34051, zebrafish, p53, WNT

## Abstract

Histone deacetylase 8 (HDAC8), a class I HDAC that modifies non-histone proteins such as p53, is highly expressed in different hematological neoplasms including a subtype of acute myeloid leukemia (AML) bearing inversion of chromosome 16 [inv(16)]. To investigate HDAC8 contribution to hematopoietic stem cell maintenance and myeloid leukemic transformation, we generated a zebrafish model with Hdac8 overexpression and observed an increase in hematopoietic stem/progenitor cells, a phenotype that could be reverted using a specific HDAC8 inhibitor, PCI-34051 (PCI). In addition, we demonstrated that AML cell lines respond differently to PCI treatment: HDAC8 inhibition elicits cytotoxic effect with cell cycle arrest followed by apoptosis in THP-1 cells, and cytostatic effect in HL60 cells that lack p53. A combination of cytarabine, a standard anti-AML chemotherapeutic, with PCI resulted in a synergistic effect in all the cell lines tested. We, then, searched for a mechanism behind cell cycle arrest caused by HDAC8 inhibition in the absence of functional p53 and demonstrated an involvement of the canonical WNT signaling in zebrafish and in cell lines. Together, we provide the evidence for the role of HDAC8 in hematopoietic stem cell differentiation in zebrafish and AML cell lines, suggesting HDAC8 inhibition as a therapeutic target in hematological malignancies. Accordingly, we demonstrated the utility of a highly specific HDAC8 inhibition as a therapeutic strategy in combination with standard chemotherapy.

## Introduction

Acute myeloid leukemia (AML) is a group of heterogeneous malignant hematological disorders underlain by genetic and epigenetic changes in hematopoietic stem cells (HSCs) and myeloid progenitors causing an imbalance between survival, proliferation and differentiation. The net effect of all changes is the accumulation of unfunctional myeloid cells, termed blasts, in the bone marrow. AML is the most frequent acute leukemia type in adults and, currently, it is curable in 35–40% of patients under 60 years of age and only in 5–15% of patients older than 60 years (Döhner et al., 2015).

Histone deacetylase 8 (HDAC8) is a ubiquitously expressed class I HDAC (Buggy et al., 2000; Hu et al., 2000; Van Den Wyngaert et al., 2000). Unlike other class I HDACs, it localizes both in the nucleus and in the cytoplasm (Li et al., 2014), lacks the C-terminal protein-binding domain (Somoza et al., 2004) and is characterized by a peculiar negative regulation of its activity by cAMP-dependent protein kinase (PKA) (Lee et al., 2004), which suggests a functional specialization. HDAC8 has been demonstrated to target non-histone proteins, such as the structural maintenance of chromosome 3 (SMC3) cohesin protein, retinoic acid induced 1 (RAI1) and p53, thus regulating diverse processes (Deardorff et al., 2012; Wu et al., 2013; Olson et al., 2014). *HDAC8* is either overexpressed or dysregulated in cancers, such as neuroblastoma, breast cancer, colon cancer (Nakagawa et al., 2007; Oehme et al., 2009; Park et al., 2011) and hematological malignancies. In particular, *HDAC8* expression was found to be increased in primary cells from childhood acute lymphoblastic leukemia patients (Moreno et al., 2010), in adult T cell leukemia/lymphoma (Higuchi et al., 2013) and human myeloma cell lines (Mithraprabhu et al., 2014). HDAC8 was demonstrated to interact with CBFβ-SMMHC fusion protein, resulting from the inversion of chromosome 16 [inv(16)] (Durst et al., 2003). The interaction of both HDAC8 and p53 with inv(16) fusion protein leads to increased deacetylation and consequent inhibition of p53, which promotes survival and proliferation of inv(16)+ AML CD34+ cells (Qi et al., 2015). Interestingly, high *HDAC8* expression was detected not only in inv(16)+ AML CD34+ cells, but also in non-inv(16)+ AML CD34+ cells, suggesting a more general involvement of HDAC8 in AML development (Qi et al., 2015). The role of HDAC8 in AML onset is further supported by a recent finding of it playing a crucial role in maintaining long-term HSC under stress condition by inhibiting p53 (Hua et al., 2017).

Histone deacetylase inhibitors (HDACi) possess an anti-cancer activity through the induction of apoptosis and cell cycle arrest (Eckschlager et al., 2017) in solid and hematological tumors (Ceccacci and Minucci, 2016; Imai et al., 2016). However, the use of HDACi is still limited due to the safety issues as side effects, including fatigue, diarrhea and thrombocytopenia, have been observed following their administration (Subramanian et al., 2010). Such toxicity is most likely related to the lack of selectivity of most of these drugs that act as pan-HDACi. In order to improve the outcome of the therapy and reduce side effects, compounds targeting specific HDAC isoforms are needed.

The distinctive structure of HDAC8, in comparison to others class I HDAC family members, allowed the development of high specific HDAC8 inhibitor PCI-34051 (hereafter PCI) (Balasubramanian et al., 2008), previously tested in T-cell lymphoma (Balasubramanian et al., 2008) and AML (Qi et al., 2015). The aim of this project was to explore the feasibility of HDAC8 inhibition as a therapeutic approach in AML. To this end, we generated a zebrafish (*Danio rerio*) model for Hdac8 overexpression that displayed a hematopoietic phenotype characterized by an increase in the hematopoietic stem/progenitor cells (HSPCs) population that could be rescued by PCI treatment. In parallel, we assessed the response of AML cell lines (OCI-AML5, HL60, PLB985, THP-1, and AML193) to PCI. We observed that PCI elicits apoptosis in THP-1 cell line and in the zebrafish embryos overexpressing Hdac8, while it induces cell cycle arrest in p53-null HL60 cells, prompting a search of alternative mechanisms explaining PCI action in the absence of p53. We, thus, demonstrated an involvement of the canonical Wnt signaling. Our results suggest that selective inhibition of HDAC8 by PCI may be a valuable therapeutic approach for the treatment of AML patients.

## Materials and Methods

### Zebrafish Embryos

Zebrafish (*D. rerio*) were maintained at the University of Milan, Via Celoria 26 – 20133 Milan, Italy (Autorizzazione Protocollo n. 295/2012-A – December 20, 2012) and Cogentech s.c.a.r.l. via Adamello 16 – 20139 Milan, Italy (Autorizzazione Protocollo n. 007894 – May 29, 2018). Zebrafish strains AB, *Tg*(*CD41:GFP*), *Tg*(*TOPdGFP*) and p53^*M*214*K*^ (Dorsky et al., 2002; Berghmans et al., 2005; Lin et al., 2005) were maintained according to international (EU Directive 2010/63/EU) and national guidelines (Italian decree No 26 of the 4th of March 2014). Embryos were staged and used until 5 days post fertilization, a time windows in which zebrafish is not considered an animal model according to national guidelines (Italian decree No 26 of the 4th of March 2014). Embryos were staged as described in Kimmel et al. (1995) and raised in fish water (Instant Ocean, 0.1% Methylene Blue) at 28°C in Petri dishes, according to established techniques. Embryonic ages are expressed in hours post fertilization (hpf) and days post fertilization (dpf). To prevent pigmentation, 0.003% 1-phenyl-2-thiourea (PTU, Sigma-Aldrich, St. Louis, MI, United States) was added to the fish water. Embryos were anesthetized with 0.016% tricaine (Ethyl 3-aminobenzoate methanesulfonate salt, Sigma-Aldrich) before proceeding with experimental protocols.

### Zebrafish Microinjection and Treatment

Injections were carried out on 1- to 2-cell stage embryos. Zebrafish *hdac8* full-length mRNA was injected at the concentration of 500 pg/embryo as previously described (Bottai et al., 2019). As a control the membrane red fluorescent protein (*mrfp)* coding mRNA was injected at the same concentration. Alternatively, in double immunofluorescence staining analyses, we injected water as a control. For canonical Wnt inhibition, zebrafish *dkk1b* mRNA was injected at the concentration of 50 pg/embryo (Mazzola et al., 2019). PCI treatment were done in 24-well plates, 30 embryos/well. PCI was added to fish water at the concentration of 150 μM PCI and embryos were kept at 28°C in the dark for 24 h. Equal concentration of DMSO was used as a control.

### FACS Analysis in Zebrafish

Embryos dissociation was achieved as described in Bresciani et al. (2018). FACS analysis were performed on *Tg*(*CD41:GFP*) zebrafish embryos at 3 dpf as previously described (Ma et al., 2011; Mazzola et al., 2019). We used Attune NxT (Thermo Fisher Scientific, Waltham, MA, United States) instrument equipped with software Kaluza (Beckman Coulter, Brea, CA, United States) for the analysis. AB wild-type embryos were used to set the gate to exclude auto-fluorescence of cells. The gate for GFP low/high cells was set on control *Tg(CD41:GFP)* embryos to distinguish a GFP_*low*_ population representing around 0.2% of total cells, as previously reported (Mazzola et al., 2019), and applied to all categories analyzed.

### Immunofluorescence

Embryos were fixed overnight in 4% paraformaldehyde (Sigma-Aldrich) in PBS at 4°C. For single-color staining, we used rabbit anti-GFP 1:500 (NC9589665, Torrey Pines Biolab, Houston, TX, United States) as primary antibody and Alexa Fluor 488-conjugated goat anti-rabbit IgG 1:400 (A11008, Invitrogen Life Technologies, Carlsbad, CA, Untied States) as secondary antibody. For dual staining, we took advantage of mouse anti-GFP 1:2000 (MAB3580, Merck-Millipore, Burlington, MA, United States), rabbit anti-histone H3 (phospho S10) 1:200 (ab5176, Abcam, Cambridge, United Kingdom), and rabbit anti-cleaved caspase 3 1:100 (9664, Cell Signaling Technologies, Danvers, MA, United States) as primary antibodies and Alexa Fluor 488-conjugated goat anti-mouse IgG and Alexa 546-conjugated goat anti-rabbit IgG 1:400 (A11001 and A11010, Invitrogen Life Technologies) as secondary antibodies. Embryos were equilibrated and mounted in 85% glycerol solution in PBS and imaged using a “TCS-SP2” confocal microscope (Leica, Wetzlar, Germany), with 20× oil immersion 9 objective, 488 nm argon ion and 405 nm diode lasers. Single stack images were acquired for each sample. Images were processed using Adobe Photoshop software. Quantification was performed by using the ImageJ software. For dual staining, we counted the total number of both GFP^+^ cells and double positive cells. The percentage of double positive cells was calculated as the ratio of double positive/total GFP^+^.

### Reverse Transcription and Real-Time Quantitative PCR

Total RNA was extracted from cells or zebrafish whole embryos (at least 30 embryos) with NucleoZOL reagent (Macherey-Nagel, Düren, Germany), according to the manufacturer’s instructions and treated with RQ1 RNase-free DNase (Promega, Madison, WI, United States). cDNA was synthetized using the GoScript Reverse Transcription Kit (Promega), as specified by the manufacturer’s instructions. qPCR analyses were performed with the GoTaq qPCR Master Mix (Promega) on the Bio-Rad iQ5 Real Time Detection System (Bio-Rad, Hercules, CA, United States) and Quantum Studio 5 (Thermo Fisher). Gene expression changes were calculated with the ΔΔCt method. We used *GAPDH* for AML cells and *rpl8* and *β-actin* for zebrafish as internal control. Primer sequences are list in Supplementary Table 1.

### Cell Lines PCI Treatment

OCI-AML5, HL60, PLB985, THP-1, and AML193 cell lines were originally obtained from ATCC/DSMZ repositories and since stored at the internal cell line bank at the Department of Experimental Oncology, IEO. Cell lines undergo regular authentication and mycoplasma testing. Cells were seeded at 10^4^ cells/well in 96-well plates in 100 μl of growth medium and allowed to grow for 24 h prior to treatment commencement. PCI was dissolved in DMSO, diluted in the appropriate culture medium and added into plates, as indicated. The concentration range of PCI has been determined based on published data and ranged between 3.12 and 50 μM (Balasubramanian et al., 2008; Qi et al., 2015). Seventy-two hours later, CellTiter-Glo assay (Promega) was performed as indicated in the manufacturer’s instructions and read on GloMax (Promega) plate reader. Cells treated with DMSO (0.2% in appropriate medium) were used as a control.

### *In vitro* Proliferation

Cells were seeded in duplicate at 10^5^ cells/ml and allowed to grow for 24 h at 37°C, 5% CO_2_, 95% humidity. Then cells were treated with 50 μM PCI or with 0.2% DMSO (control). At 12, 24, 48, and 72 h, both viable and dead cells were counted under an inverted-light microscope (Leica) following 0.4% trypan blue staining.

### Cell Cycle Analysis

Cells were seeded at 10^5^ cells/ml and then treated with DMSO or with 50 μM of PCI for 48 h. One-million of viable cells were harvested after 12, 24, and 48 h, washed once with cold PBS, fixed in 70% of ice-cold ethanol dropwise and kept on ice for 30 min. Next, cells were washed in 1% BSA in PBS and stained overnight with DNA staining solution containing 250 μg/ml of RNase and 5 μg/ml propidium iodide (PI) at 4°C. Data analysis was done using flow cytometry (FACSCelesta, FlowJo10 software).

### Apoptosis Assay

Cells were seeded at 10^5^ cells/ml and then treated with DMSO or with 50 μM of PCI for 72 h. A total of 50 × 10^4^ cells were harvested after 72 h, washed once with cold PBS and then with annexin buffer. Cells were resuspended in 100 μl annexin-APC diluted 1:50 in annexin buffer and incubated 1 h at room temperature in the dark. Next, cells were washed once with annexin buffer and resuspended in 1× PBS. Cells were stained for maximum 5 min with PI solution. Data analysis was done using flow cytometry (FACSCelesta, FlowJo10 software).

### Combination Treatment

Cells were seeded at 10^4^ cells/well in 96-well plates and allowed to grow for 24 h prior to treatment commencement. Drug concentrations ranged from 0.78 to 100 μM and from 0.078 to 10 μM for PCI and cytarabine, respectively. Cells were treated with all concentrations of single agents and in combination setting, in which decreasing concentrations of each compound were used together (Supplementary Figure 5). The combination index (CI), based on the Bliss Independence model, was calculated as $\text{CI}=\frac{E_{A}+E_{B}-E_{A}\,E_{B}}{E_{A\,B}}$, where E_*A*_ indicates the effect of compound A, E_*B*_ indicates the effect of compound B and E_*AB*_ the effect of the combination of both compounds. CI < 1 indicates synergism; CI = 1 indicates an additive effect, while CI > 1 indicates antagonism (Foucquier and Guedj, 2015).

### Statistical Analysis

Each experiment was performed at least twice (biological replicates). A minimum number of 15 embryos was analyzed in each imaging experiment, while RNA was extracted from a minimum of 30 animals. PCI treatment outcome was assessed on at least 30 zebrafish embryos. For qPCR analysis on zebrafish, experiments were performed on at least three different independent experiments (batches of embryos deriving from different matings). The statistical significance was determined using two-sided Student’s *t*-test when comparing two groups and one-way ANOVA test followed by Tukey *post hoc* correction when comparing three groups. One-sample *t*-test was used when control group was set to a defined value of 1. Data were considered significant if *p* < 0.05.

## Results

### Hdac8 Overexpression in Zebrafish Leads to HSPCs Expansion and Its Inhibition Elicits Apoptosis and Rescues the Phenotype

**FIGURE 1:**
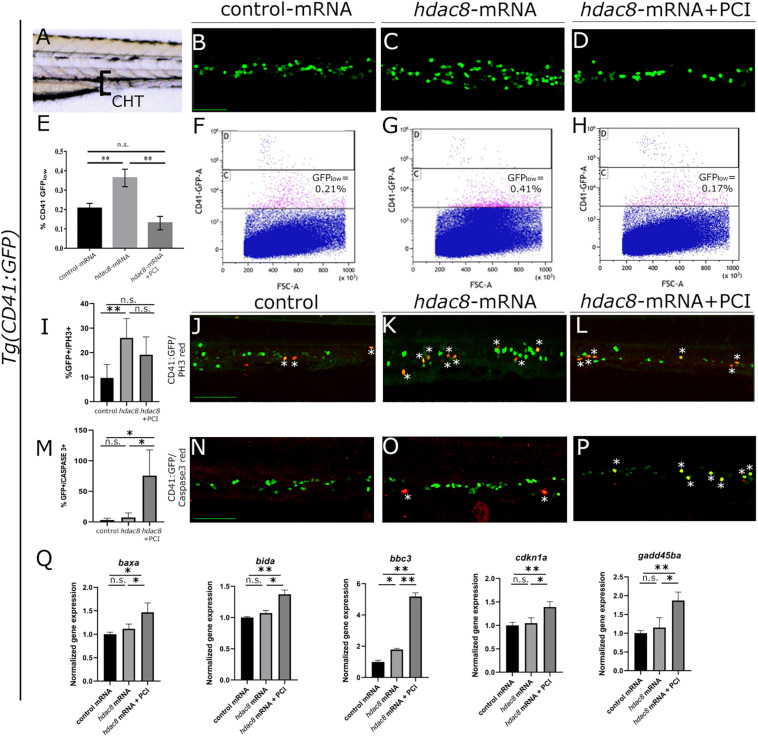
Analysis of Hdac8 overexpression in zebrafish and apoptosis induced by PCI treatment. **(A)** Scheme of trunk-tail region of zebrafish embryos: confocal imaging was always performed on the same embryo region, comprising the tip of the yolk sack extension, between the dorsal aorta and the vein; representative images of **(B)** control; **(C)** *hdac8*-mRNA injected; **(D)** PCI-treated *hdac8*-mRNA injected *Tg(CD41:GFP*) zebrafish embryos at 3 dpf. Scale bar represents 100 μm. **(E)** Quantification by FACS of GFP_*low*_-HSPCs of **(F)** control; **(G)** *hdac8*-mRNA injected; **(H)** PCI-treated *hdac8*-mRNA injected *Tg(CD41:GFP*) zebrafish embryos at 3 dpf. The results are presented as mean ± SD from three independent experiments. **(I–L)** Proliferation of HSPCs in the caudal hematopoietic tissue of the *Tg(CD41:GFP)* zebrafish line following Hdac8 overexpression and PCI treatment. GFP^+^ HSPCs in green; PH3 in red; quantification in **(I)**, *N* = 6 embryos analyzed. **(M–P)** Apoptosis of HSPCs in the caudal hematopoietic tissue of the *Tg(CD41:GFP)* zebrafish line following PCI treatment. GFP^+^ HSPCs in green; activated caspase-3^+^ cells in red; quantification in **(M)**, *N* = 3 embryos analyzed. Asterisks indicate double positive cells (yellow). Scale bar represents 100 μm. **(Q)** RT-qPCR analyses of apoptotic markers *baxa*, *bida, bbc3, cdkn1a*, and *gadd45ba* expression in control mRNA and *hdac8*-overexpressing zebrafish embryos treated with DMSO or PCI. The results are presented as mean value ± SD from three independent experiments. *p < 0.05, **p < 0.01, n.s. = not significant, one-way ANOVA followed by Tukey *post hoc* correction.

CD34+ cells derived from inv(16)+ AML patients express high levels of HDAC8 (Qi et al., 2015). We generated a zebrafish model for Hdac8 overexpression by injecting embryos with the full-length zebrafish *hdac8* mRNA (500 pg/embryo) to assess whether HDAC8 upregulation would alter hematopoietic phenotype *in vivo*. The injection of the *hdac8*-mRNA, although increasing the *hdac8* transcript and protein levels (Supplementary Figures S1A,B), did not alter the general morphology of the embryo or organ size (Supplementary Figures S1C–E) but specifically impact on the hematopoietic phenotype. In this regard, to obtain an easy read-out of the hematopoietic phenotype, we performed *hdac8* overexpression in the *Tg*(*CD41:GFP*) transgenic line that expresses GFP protein in HSPCs, Lin et al. (2005); Supplementary Figures S1F,G, and we assessed the expression of the HSC transcription factor markers *c-Myb*, *gata2b* and Runx1, and of the immature myeloid cells *pu.1* that resulted upregulated following Hdac8 ectopic expression (Supplementary Figures 1H,I). Confocal images of the caudal hematopoietic tissue (CHT) in 3 dpf embryos (Figure 1A), showed an increase in HSPC population upon Hdac8 overexpression in comparison to controls (Figures 1B-C). This effect was specific, as we obtained a significant reduction of HSPCs in *hdac8* mRNA-injected embryos treated with PCI (Figure 1D). We then quantified HSPC number by enumerating GFP_*low*_-HSPCs in the three categories of embryos by flow cytometry (Ma et al., 2011; Mazzola et al., 2019). GFP_*low*_-HSPCs were significantly increased in *hdac8*-injected embryos compared to controls and were reduced when treated with PCI (Figures 1E–H). We also evaluated the effect of PCI on zebrafish HSPCs in the absence of Hdac8 overexpression as a control. Immunofluorescence and FACS analyses for the HSPCs in the *Tg*(*CD41:GFP*) line and gene expression analyses for the HSC marker *cmyb* indicated a decrease of HSPCs in PCI-treated embryos compared to control embryos (Supplementary Figures 2A–F). To assess whether the expansion in HSPCs population following Hdac8 ectopic expression is indicative to a pre-leukemic state and if these cells possess higher self-renewal ability compared to their differentiated counterparts, we performed dual immunofluorescence with GFP (in green) and phospho-histone H3 (PH3, in red) in the *Tg(CD41:GFP)* embryos at 3 dpf. The ectopic expression of Hdac8 induced an increased proliferation of HSPCs in comparison to controls, that was reduced following PCI treatment (Figures 1I–L).

Since p53 is a target of HDAC8 and studies indicate that PCI treatment determines cell cycle arrest and induction of apoptosis in *in vivo* (Oehme et al., 2009) and *in vitro* (Rettig et al., 2015) models, we verified whether PCI treatment determines apoptosis also in zebrafish (Supplementary Figures 2G,H). Thus, to assess PCI-mediated apoptosis specifically in the HSPC population which was expanded following *hdac8*-overexpression, we evaluated caspase-3 activation by dual immunofluorescence in Hdac8-overexpressing *Tg(CD41:GFP)* zebrafish embryos at 3 dpf treated or not with PCI. We observed an increase of caspase-3^+^/GFP^+^ HSPCs in PCI-treated *hdac8*-mRNA injected embryos compared to *hdac8*-mRNA-injected and control embryos (Figures 1M–P). This result was confirmed by the significant increase of expression levels of the p53 target genes (*baxa, bida, bbc3, cdkn1a*, and *gadd45ba*) by RT-qPCR in PCI-treated *hdac8*-mRNA injected embryos compared to both control mRNA- and *hdac8*-mRNA-injected embryos (Figure 1Q; Qi et al., 2015). Taken together, these results indicate that Hdac8 overexpression in zebrafish determines an expansion of HSPC population and that PCI treatment induces a block in cell expansion activating p53-mediated apoptosis.

### PCI Exerts Cytostatic and Cytotoxic Effect on AML Cell Lines

**FIGURE 2:**
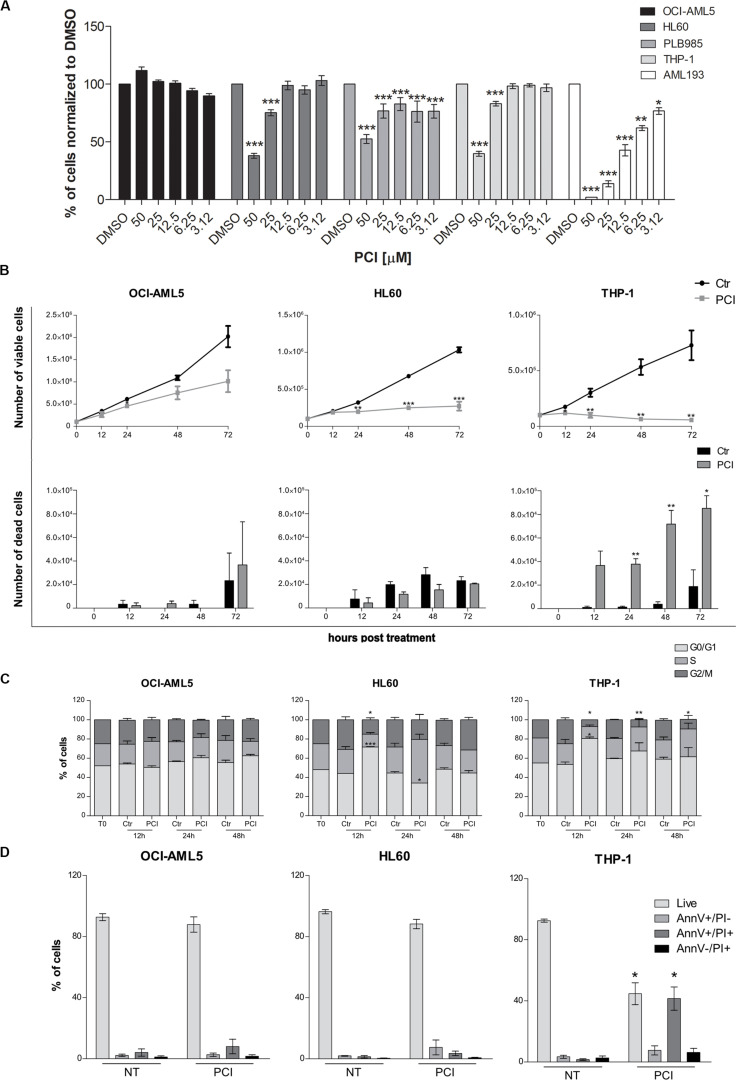
Cytostatic and cytotoxic effect of PCI in AML cell lines. **(A)** The indicated cell lines were treated for 72 h with different concentration of PCI, or DMSO alone as a control. CTG assay was used to assess the effect of the treatment on the cell viability. The results are presented as mean ± SD from four technical replicates deriving from one independent experiment for PLB985 and AML193 cell lines, and at least two independent experiments for OCI-AML5, HL60, and THP-1 cell lines. **(B)** Effect of PCI on AML cell line growth. Cells were treated with 50 μM of PCI or with DMSO as a control. Cell viability (upper panel) and cell death (lower panel) were determined 12, 24, 48, and 72 h after treatment using trypan blue staining. The results are presented as mean ± SEM from two independent experiments for 12 h and three independent experiments for the others time points. **(C)** Cell cycle time course over 48 h of PCI treatment. Cells were treated with 50 μM of PCI or with DMSO as a control. Histogram showing cell distribution in three different cell cycle phases indicated as diverse shades of gray. The results are presented as mean ± SEM from two independent experiments. **(D)** Induction of apoptosis upon 72 h of PCI treatment. Cells were treated with 50 μM of PCI or with DMSO as a control. Histogram showing the percentage of live, AnnV+/PI, AnnV+/PI+ and AnnV–/PI+ cells indicated as diverse shades of gray. The results are presented as mean ± SEM from three independent experiments. NT, untreated; T0, time zero; AnnV, annexin V; PI, propidium iodide. *p < 0.05, **p < 0.01, ***p < 0.001, Student’s *t*-test.

To evaluate the effects of HDAC8 inhibition also in human myeloid cells, we selected five AML cell lines expressing *HDAC8* (OCI-AML5, HL60, PLB985, THP-1, and AML193). We treated them once for 72 h with decreasing concentrations of PCI and evaluated the viability using CTG luminescence assay, an indicator of metabolically active cells. PCI decreased the viability of HL60, PLB985, THP-1, and AML193 cell lines while OCI-AML5 seemed less sensitive to the treatment (Figure 2A).

We excluded from further analyses the PLB985 cell line since it is a sub-clone of HL60 and AML193 childhood AML cell line as all the other cell lines derive from adult AML patients. We validated HDAC8 expression levels by means of RT-qPCR and western blot analyses. HL60 and THP-1 expressed significantly higher *HDAC8* mRNA than OCI-AML5 by RT-qPCR (Supplementary Figure 3A). Western blot analysis also indicated higher HDAC8 protein levels in HL60 and THP-1 than in OCI-AML5 (Supplementary Figure 3B). To determine whether PCI elicits cytostatic of cytotoxic effect, we treated the selected cell lines with 50 μM of PCI or with DMSO as a control for 12, 24, 48, and 72 h and counted daily following trypan blue staining. No significant effect on cell growth was observed in the less-PCI-sensitive OCI-AML5 cell line (Figure 2B), although PCI treatment increased the levels of acetylated SMC3 (ac-SMC3), a specific HDAC8 target (Supplementary Figure 3C). By contrast, PCI exerted a cytostatic effect in HL60 cell line and caused cell death in THP-1 cells, as indicated by viable and dead cell count (Figure 2B), which spurred us to investigate how PCI treatment impacted cell cycle. To this end, we treated AML cell lines with 50 μM PCI for 12, 24, and 48 h and analyzed DNA content by PI staining. At 12 h PCI treatment resulted in a block in the G0–G1 phase of over 70 and 80% of HL60 and THP-1 cells, respectively, while we did not detect any variations between untreated and treated OCI-AML5 samples (Figure 2C). Cell cycle arrest following PCI treatment in HL60 and THP-1 cell lines was underlain by a decrease in *CyclinD1* (*CCND1*) and *CMYC* expression (Supplementary Figure 4). Next, we evaluated apoptosis induction using Annexin V/PI staining and we demonstrated that PCI treatment induces apoptosis only in THP-1 cells as attested by increased percentages of Annexin V^+^/PI^+^ positive population corresponding to late apoptotic cells at 72 h. Indeed, the HL60 cells responded to PCI inhibition but cannot undergo apoptosis lacking functional p53 (Wolf and Rotter, 1985; Figure 2D).

Taken together, these data indicate that PCI treatment impacts on AML cell survival causing cell cycle arrest followed by apoptosis when p53 is functional.

### PCI Synergizes With Cytarabine in AML Cell Lines

**FIGURE 3:**
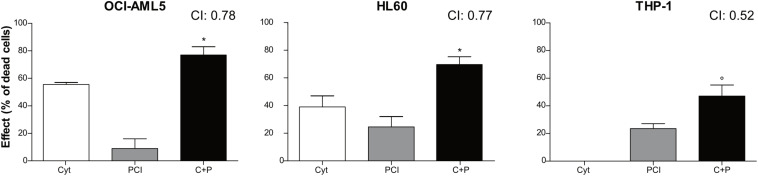
Combination treatment with cytarabine + PCI. OCI-AML5, HL60, and THP-1 cell lines were treated for 72 h with single compound and their combination. CTG assay was used to assess the effect of each treatment on the cell culture viability. The bar graph represents the effect of one of the eight concentrations tested. CI values are shown for each combination. The results are presented as mean ± SEM from two independent experiments. Asterisk (*) indicates significance of combination treatment versus PCI, circle (°) indicates significance of combination treatment versus cytarabine. One symbol, *p* < 0.05, Student’s *t*-test. Cyt and C, cytarabine; P, PCI; CI, combination index.

Combination therapy allows for dose reduction, lowers the incidence and severity of side effects and prevents the development of resistance. We treated AML cell lines with cytarabine, an agent used at the frontline of AML treatment, alone or together with PCI to assess whether their combination resulted in synergy, additivity or antagonism as an *in vitro* indicator of a potential advantage of combination over single-agent treatment. OCI-AML5, HL60, and THP-1 cells were treated for 72 h with cytarabine at concentrations ranging from 0.078 to 10 μM and PCI at concentration range of 0.78–100 μM alone or together mixing decreasing concentrations of each compound (Supplementary Figure 5). Based on CI, a synergistic effect was observed for all AML cell lines when combining 0.35 μM cytarabine with 25 μM PCI. The combination of cytarabine and PCI resulted in an effect that was greater than the sum of single treatment outcomes in OCI-AML5 and HL60 cell lines, whilst showing a dramatic combination effect in THP-1 cell line at a concentration of cytarabine that alone had no effect. In detail, in THP-1 cell line the combination resulted in 47% of cell death compared to 0% of cytarabine and 23.5% of PCI in single-agent setting (Figure 3).

These results might suggest that cytarabine doses can be reduced in combination therapy while eliciting the same inhibitory effect on cell proliferation.

### HDAC8 Inhibition Downregulates Canonical Wnt Pathway

**FIGURE 4:**
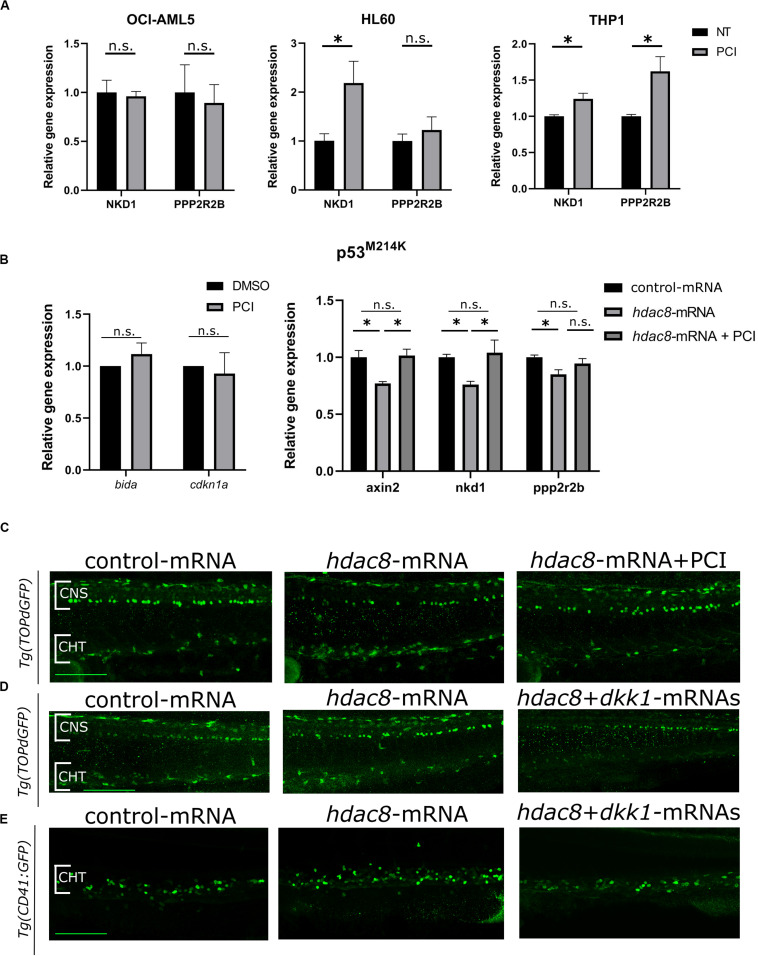
Canonical Wnt pathway modulation by HDAC8. **(A)** Canonical Wnt pathway modulation by PCI in OCI-AML5, HL60, and THP-1 cell lines. *NKD1* and *PPP2R2B* Wnt inhibitors were analyzed by RT-qPCR. Results are presented as mean ± SD from three independent experiments. *p < 0.05, Student’s *t*-test. **(B)** WNT pathway modulation by PCI in zebrafish mutant p53^*M214K*^. *bida* and *cdkn1a* apoptotic genes and *axin2*, *nkd1* and *pppr2r2b* WNT target genes were analyzed by RT-qPCR. Results are presented as mean ± SD from three independent experiments. n.s.: not significant, *p < 0.05; One sample *t*-test for apoptotic genes, one-way ANOVA followed by Tukey *post hoc* correction for canonical WNT pathway genes. **(C)** Representative images of canonical Wnt modulation by *hdac8* overexpression and PCI on HSPCs in the CHT of the Wnt reporter line *Tg(TOPdGFP)*. **(D,E)** Representative images of canonical Wnt modulation by *hdac8* overexpression and Wnt inhibition by *dkk1b* injection on HSPCs in the CHT of the Wnt reporter line *Tg(TOPdGFP)* and of the HSPCs reporter line *Tg(CD41:GFP)*. NT, not treated; CNS, central nervous system; CHT, caudal hematopoietic tissue. Scale bar represents 100 μm.

We wished to identify a mechanism of action responsible for PCI-induced growth arrest in HL60 cells that responded to PCI inhibition without undergoing apoptosis being p53-null. Recently, we and others demonstrated that HDAC8 activates canonical Wnt pathway (Tian et al., 2015; Ferrari et al., 2019), which is frequently dysregulated in AML (Gruszka et al., 2019). We investigated whether Wnt signaling was affected by HDAC8 inhibition in AML cell line. We analyzed by RT-qPCR the expression levels of canonical Wnt pathway inhibitors *NKD1* and *PPP2R2B*, previously reported to be downregulated by forced *HDAC8* expression and upregulated following PCI treatment (Tian et al., 2015). We observed that the expression levels of both *NKD1* and *PPP2R2B* increased following PCI treatment in PCI sensitive cell lines HL60 and THP-1, indicating that HDAC8-mediated downregulation of the canonical Wnt signaling could be the cause of cell cycle arrest in these cell lines. Interestingly, the Wnt pathway was not modulated in the less-sensitive OCI-AML5 cells, confirming the specificity of HDAC8 inhibition on Wnt regulation (Figure 4A).

To verify that the downregulation of the Wnt pathway is p53-independent, we assessed Wnt pathway modulation in p53 mutant zebrafish embryos, which mimicked the p53-null HL60 condition. Thus, we took advantage of a homozygous zebrafish p53^*M*214*K*^ mutant line, which lacks functional p53 (Berghmans et al., 2005), and we observed that, although the expression of the apoptotic markers *bida* and *cdkn1a* was not affected as expected in a p53 null background, a decrease of the expression of Wnt inhibitors *axin2*, *nkd1*, and *ppp2r2b* was observed in Hdac8*-*overexpressed mutant embryos compared to controls while PCI treatment restored the expression of Wnt inhibitors (Figure 4B). To further demonstrate that Hdac8 modulates Wnt signaling, we evaluated the regulation of canonical Wnt signaling by assessing the levels of active- and total-β catenin by western blot techniques (Supplementary Figure 6), and we used a zebrafish canonical Wnt reporter transgenic line *Tg(TOPdGFP)* (Dorsky et al., 2002). Following *hdac8* overexpression, canonical Wnt signaling was increased also in the HSPCs in the CHT region, while it was switched off following PCI administration (Figure 4C and Supplementary Figure 7). Interestingly, a similar reduction in the HSPCs in the CHT region of *Tg(TOPdGFP)* or *Tg(CD41:GFP)* zebrafish embryos was achieved following inhibition of the canonical Wnt signaling in *hdac8*-mRNA-injected embryos by means of co-injection of the *dkk1b* transcript (50 pg/embryo) (Mazzola et al., 2019; Figure 4D,E and Supplementary Figure 7). Taken together, these data demonstrate that HDAC8 activates canonical Wnt pathway that, in turns, regulates hematopoietic cell proliferation (Richter et al., 2017; Mazzola et al., 2019). PCI administration downregulates Wnt signaling and reduces HSPCs, an important finding as Wnt downregulation is a clinical treatment currently in use for AML patients.

## Discussion

Aberrations in epigenetic regulators contribute to cancer, including leukemia insurgence, hence, the use of epigenetic modifiers may comprise a promising therapeutic approach (Nakagawa et al., 2007; Zhang et al., 2012). Epigenetic defects are generally reversible, as opposed to genetic changes, providing a strong rationale for a pharmaceutical intervention. Low level of acetylation due to high expression of HDACs (Nakagawa et al., 2007; Wang et al., 2016) is one of the most frequent epigenetic modifications found in cancer cells. HDACs are more expressed in hematological malignancies including AML than in normal hematopoietic cells (Bradbury et al., 2005; Marquard et al., 2009), and we demonstrated that forced expression of Hdac8 in zebrafish embryos induced an increase in HSPC number that can be rescued with the use of a specific HDAC8 inhibitor. HDACi have been used as therapeutic agents in AML, myelodysplastic syndromes, lymphoma, and chronic lymphoblastic leukemia (Melnick and Licht, 2002; Altucci and Minucci, 2009; Gloghini et al., 2009); however, monotherapy elicits modest effects. There are several possible reasons behind this failure. For example, HDACi exert different outcomes depending on timing of administration and differentiation stage of the tumor (Kuendgen et al., 2011; Garcia-Manero et al., 2012; Novotny-Diermayr et al., 2012; Romanski et al., 2012; Xie et al., 2012; Candelaria et al., 2017; Huang and Zong, 2017; Young et al., 2017). In addition, the vast majority of preclinical and clinical studies deploying HDACi for anti-cancer treatment involved unselective inhibitors targeting all HDACs (pan-HDACi) with broad spectrum of side effects and toxicity, thus calling for exploitation of agents that specifically block individual HDACs. PCI is a specific small molecule inhibitor endowed with 200-fold higher selectivity for HDAC8 than for other HDACs; it is more effective and less toxic than pan-HDACi (Balasubramanian et al., 2008).

We demonstrated that HSPCs are sensitive to selective HDAC8 inhibition both in a zebrafish embryological context as well as in adult derived-AML cell lines. This is in agreement with findings of Qi et al. (2015) that showed that leukemic cells bearing inv(16) linked to high expression levels of HDAC8 (5–12 times that of CD34+ cells from healthy donors) were particularly responsive to treatment with PCI, although also non-inv(16) AML blasts showed a degree of sensitivity. Since the hierarchy of HSPCs is finely tuned during development until adulthood and subjected to different regulatory cues, our demonstration that the HDAC8 inhibition is effective on HSPCs during embryogenesis and in the adult, provides a common mechanism against HSPCs self-renewal and amplification and an attractive therapeutic treatment for the future.

We found that PCI elicits cytostatic or cytotoxic effect in AML sensitive cell lines. Mechanistically, sensitive cells undergo cell cycle arrest, followed by apoptosis when expressing p53. Cell cycle arrest in the G0/G1 phase and induction of p21WAF1/CIP1 expression was previously observed in neuroblastoma cells upon HDAC8 silencing, while an increase of cells in G2/M phase of the cell cycle was reported in hepatocellular cancer cells treated with PCI (Tian et al., 2016). p53 is a known HDAC8 target and its aberrant deacetylation by HDAC8 disables p53 function and promotes leukemic transformation (Wu et al., 2013). HDAC8 knockout or pharmacological inhibition effectively restores p53 acetylation and activity inducting apoptosis in inv(16)^+^AML CD34^+^ cells (Qi et al., 2015). Similarly, we observed p53-dependent apoptosis specifically in CD41-GFP^+^ hematopoietic compartment following PCI treatment of zebrafish embryos overexpressing Hdac8. This population was increased in zebrafish embryos upon Hdac8 overexpression. Our findings are consistent with literature data demonstrating that HDAC8 regulates HSPC survival under hematopoietic stress by modulating p53 activity (Liu et al., 2009; Asai et al., 2011, 2012). Our experiments show that THP-1 cells underwent apoptosis, while the p53-null HL60 cell line remained blocked in the G0/G1 phase of the cell cycle. This led us to consider alternative mechanisms of growth arrest elicited by PCI treatment. Studies show that canonical WNT signaling is activated by HDAC8 (Tian et al., 2015; Ferrari et al., 2019). We now show that canonical Wnt pathway is significantly downregulated both in cell lines and in zebrafish embryos following HDAC8 inhibition. Downregulation of canonical Wnt pathway by PCI has been described in a model of hepatocellular cancer, in which HDAC8 physically interacts with chromatin modifier EZH2 to repress Wnt antagonists, activating Wnt pathway. PCI treatment, instead, reduced active β-catenin and *cyclin D1* expression in this system. We showed that PCI downregulates Wnt signaling independently of p53 status; however, it does not kill cells unless p53 is functional (Tian et al., 2016).

We explored the possibility of performing combination treatment and combined cytarabine with PCI. The two compounds synergize in all cell lines treated, including the less-PCI-sensitive OCI-AML5 cells. Although we failed to reduce the concentration of PCI, in THP-1 cell line the synergy was observed when combining a dose of cytarabine that alone elicits no effect. This may indicate that AML patients with high HDAC8 and functional p53 may particularly benefit from this combination. Indeed, a recent study reported the efficacy of HDAC8 inhibition in combination with FLT3 inhibitor in suppressing FLT3-ITD^+^ AML cells, thus sustaining the potential of combination treatment employing HDACi and standard chemotherapy (Long et al., 2020). Phase II and III clinical trial results confirm that HDACi act more efficiently when combined with conventional chemotherapy. However, more studies are needed to understand the precise mechanism of action of the combination.

Taken together, our study validates the preclinical potential of specific inhibition of HDAC8 as a potent therapeutic approach in AML.

## Data Availability Statement

The original contributions presented in the study are included in the article/Supplementary Material, further inquiries can be directed to the corresponding authors.

## Author Contributions

AP, AM, CB, AG, and MA conceived and designed the experiments. MS, MM, and GD performed the experiments on zebrafish. AG and DV performed the experiments on AML cells. AQ and MT performed the FACS analyses on zebrafish. MS, MM, GD, and AP analyzed the data on zebrafish. AG, DV, and CB analyzed the data on AML cells. MS, AG, DV, and AP wrote the manuscript. AP, AM, and AG supervised the manuscript drafting. AP supervised the research project. All authors contributed to the article and approved the submitted version.

## Conflict of Interest

GD, AQ, and MT was employed by company Cogentech. The remaining authors declare that the research was conducted in the absence of any commercial or financial relationships that could be construed as a potential conflict of interest.
