# Supplementary material for: HDAC8: A Promising Therapeutic Target for Acute Myeloid Leukemia
Source: Front Cell Dev Biol. 2020 Sep 4;8:844. doi: 10.3389/fcell.2020.00844 (PMC7498549; doi:10.3389/fcell.2020.00844)
Supplement: Supplementary file 1 [file Data_Sheet_1.pdf]

Supplementary Material

1 Supplementary Methods

1.1 Western blot

Total proteins from zebrafish embryos (at least 30 embryos) were extracted with Laemmli buffer with the addition of protease inhibitor cocktail (Roche), 2 µl/embryo. Lysates were incubated 3 min at 95°C and 2 min at 4°C, followed by disaggregation by using insulin syringe. Incubation and disaggregation were repeated twice and then lysates were centrifuged 10 min at 16.000 g at 4°C. The supernatant was recovered and extracts were quantified by using the Quantum Micro protein Assay (EuroClone). 40 µg of proteins were loaded in a 10% acrylamide/polyacrilammide gel and subjected to electrophoresis. Total proteins from AML cell lines were extracted with Laemmli sample buffer. 20 µl of extracts were loaded in a 10% acrylamide/polyacrilammide gel and subjected to electrophoresis. Proteins were transferred onto polyvinylidene fluoride (PVDF) membranes that were incubated with blocking solution (BS) (5% skimmed powder milk in TBS containing 0.1% TWEEN-20) for 1h at room temperature before overnight incubation at 4°C with primary antibodies in blocking solution. Membranes were then incubated 1h at room temperature with HRP-conjugated secondary antibodies in blocking solution. Protein bands were detected by using WESTAR ECL detection system (Cyanagen, Bologna, Italy). Images were acquired with the Alliance MINI HD9 AUTO Western Blot Imaging System (UVItec Limited, Cambridge, UK) and analyzed with the related software. Tubulin or vinculin were used as internal control. Antibodies are list in **Supplementary Table 2**.

2 Supplementary Figures and Tables

2.1 Supplementary Figures

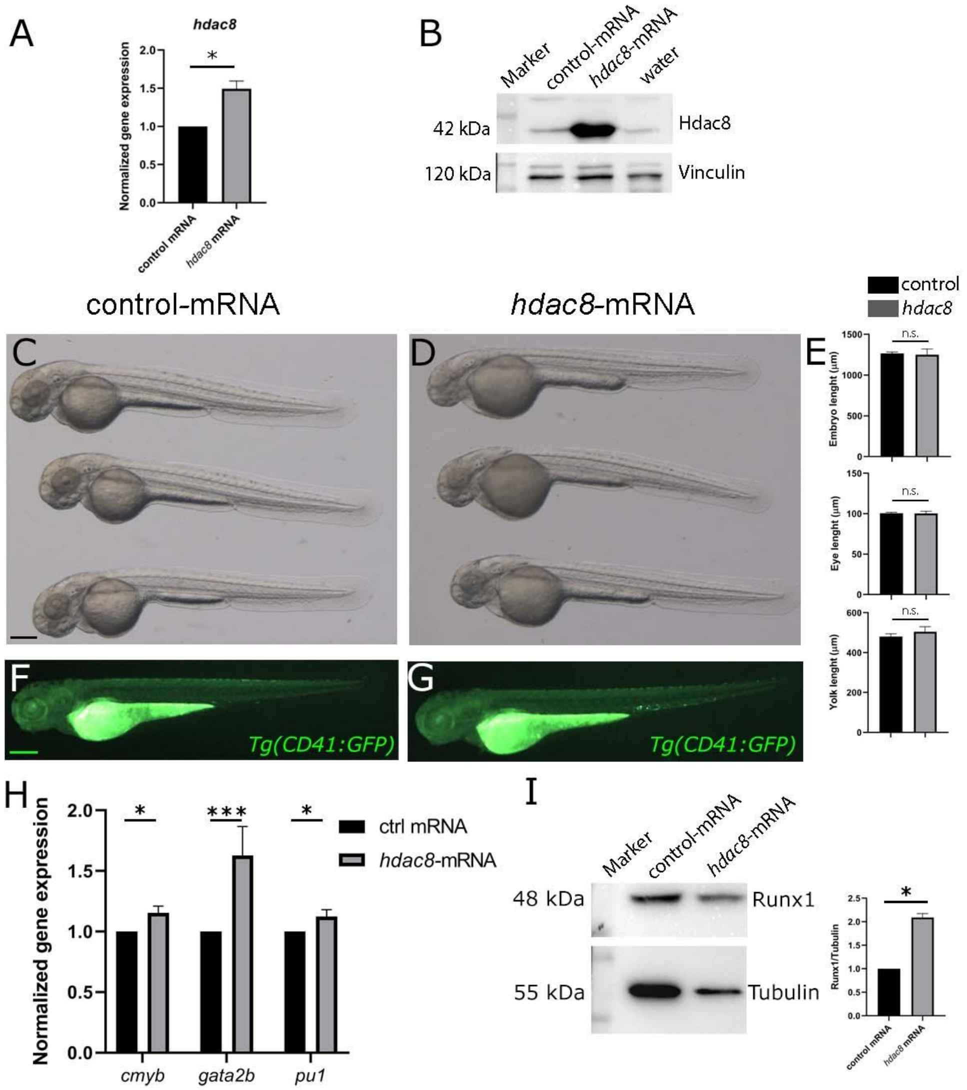

Figure S1: Effects of Hdac8 ectopic expression in zebrafish

RT-qPCR analysis **(**A) and Western blot analysis (B) of HDAC8 expression in control mRNA-, *hdac8* mRNA- and water injected embryos. The results in (A) are presented as mean value ± SD from three independent experiments; *p<0.05, One sample t test. Morphological phenotypes of control mRNA- (C) and *hdac8* mRNA- (D) injected embryos at 48 hpf. (E) Quantification of 3 dpf embryo length, eye diameter and yolk extension length (N=10). n.s.: non significative, Student's t test. Morphological phenotypes of control mRNA- (F) and *hdac8* mRNA-(G) injected embryos at 3 dpf of the *Tg(CD41:GFP)* transgenic line. Scale bar represents 100 µm. (H) *c-Myb*, *gata2b* and *pu.1* expression analysis at 48 hpf by RT-qPCR techniques. Histograms represent mean value ± SD from three independent experiments. *p<0.05, ***p<0.001, One sample t test. (I) Western blot analysis of Runx1 expression at 48 hpf. Histograms represent mean value ± SD from two independent experiments. *p<0.05, One sample t test.

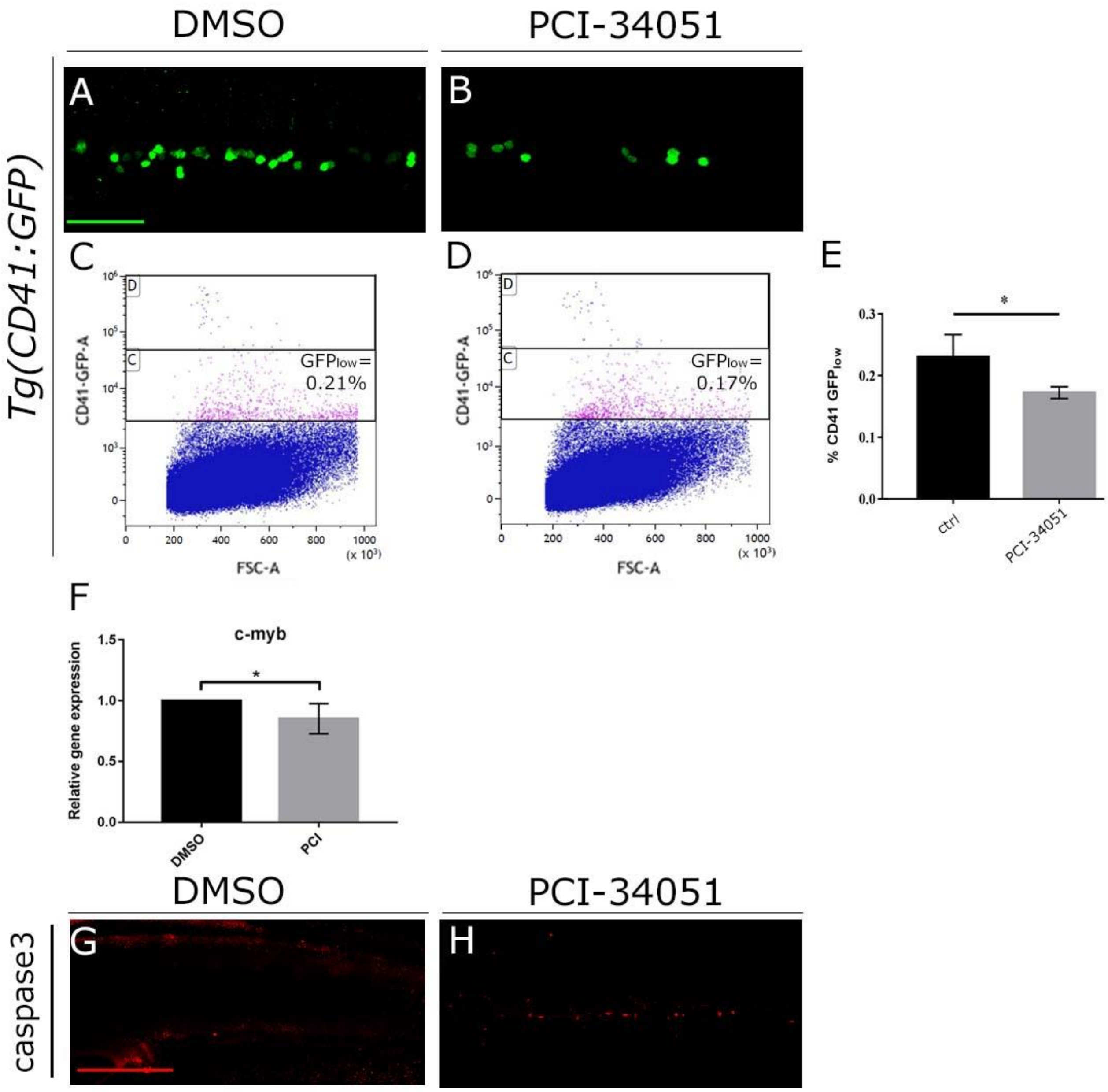

Figure S2: Effect of PCI treatment in zebrafish

(A, B) Confocal imaging of CHT of (A) DMSO or (B) PCI-treated *Tg(CD41:GFP)* zebrafish embryos at 3 dpf. (C-D) Quantification by FACS of GFP_{low}-HSPCs of (C) DMSO and (D) PCI-treated *Tg(CD41:GFP)* zebrafish embryos at 3 dpf, quantification in (E). The results in (E) are presented as mean value ± SD from four independent experiments; * $p<0.05$, Student's t test. (F) RT-qPCR quantification of the HSCs marker *c-myb* in DMSO- and PCI-treated zebrafish embryos. Histograms represent mean value ± SD from four independent experiments; *$p<0.05$, One sample t test. (G, H) Confocal imaging of CHT of (A) DMSO or (B) PCI-treated zebrafish embryos at 3 dpf stained with caspase3 antibody to detect apoptosis. Scale bar in A, B, G, H represents 100 µm.

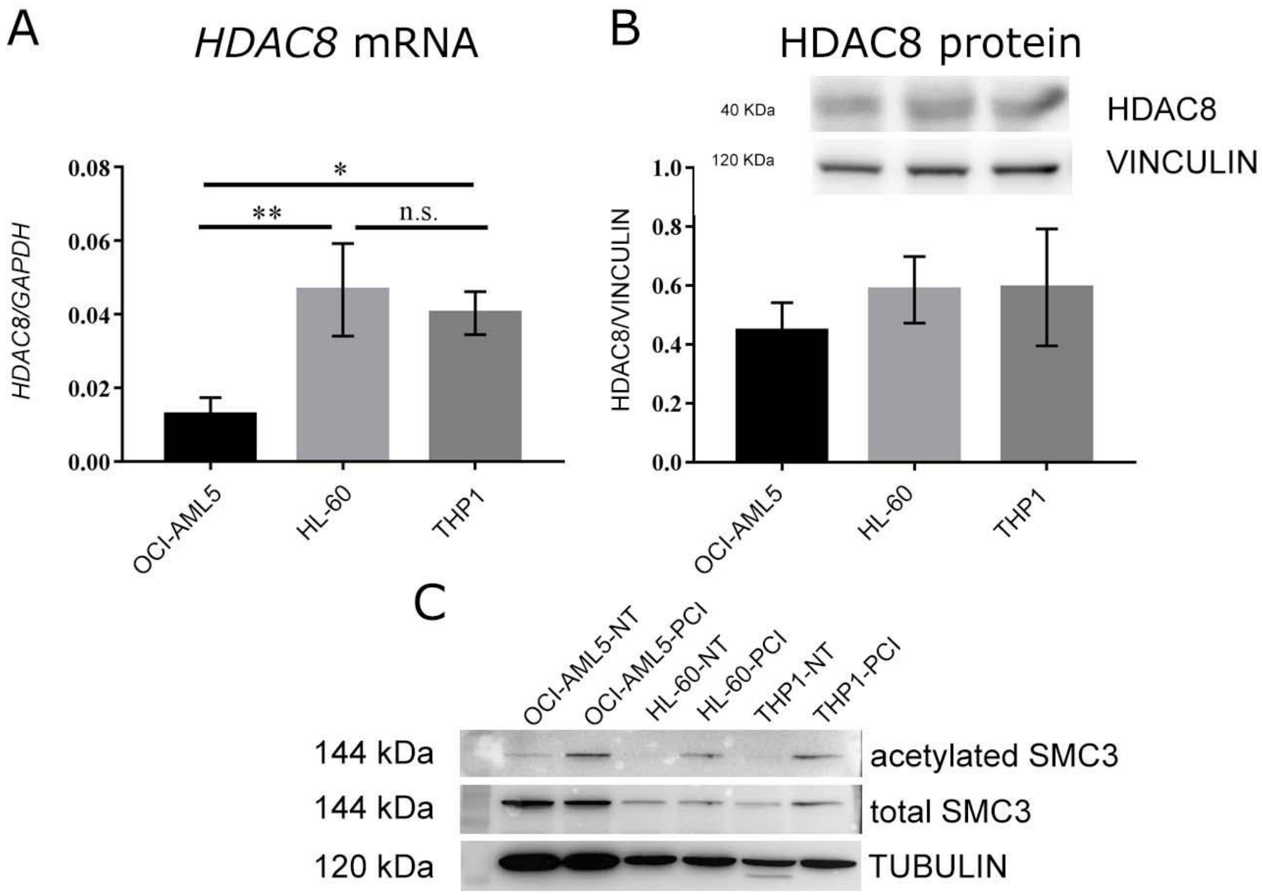

Figure S3: Assessment of HDAC8 expression and PCI efficacy in OCI-AML5, HL-60 and THP1 AML cell lines.

RT-qPCR analysis (A) and Western blot analysis (B) of HDAC8 expression. The results are presented as mean value ± SD from three independent experiments; *p<0.05, **p<0.01, ns: not significant, ANOVA One-Way followed by Tukey post-hoc correction. (C) Western blot analyses of SMC3 acetylation status in control untreated (NT) and PCI–treated AML cell lines.

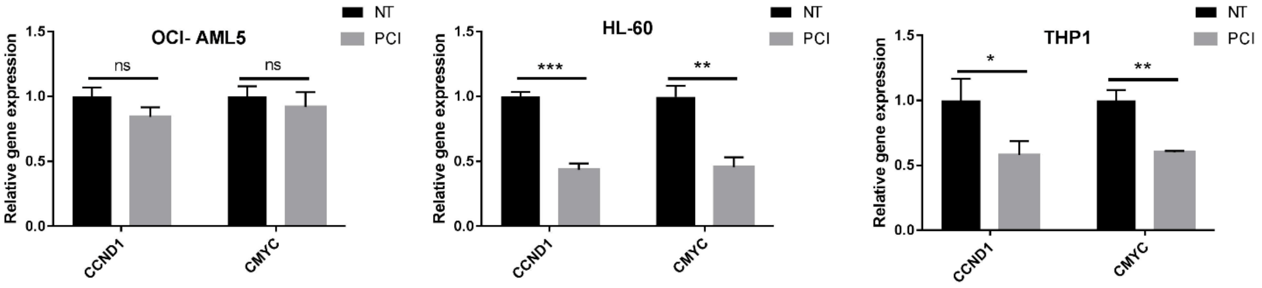

Figure S4: PCI blocks cell cycle progression in AML cell lines
RT-qPCR analyses of *CCND1* and *CMYC* gene expression in control untreated (NT) and PCI-treated OCI-AML5, HL-60 and THP1 AML cell lines. The results are presented as mean value ± SD from three independent experiments. ns: not significant, *p<0.05, **p<0.01, ***p<0.001; Student's t test.

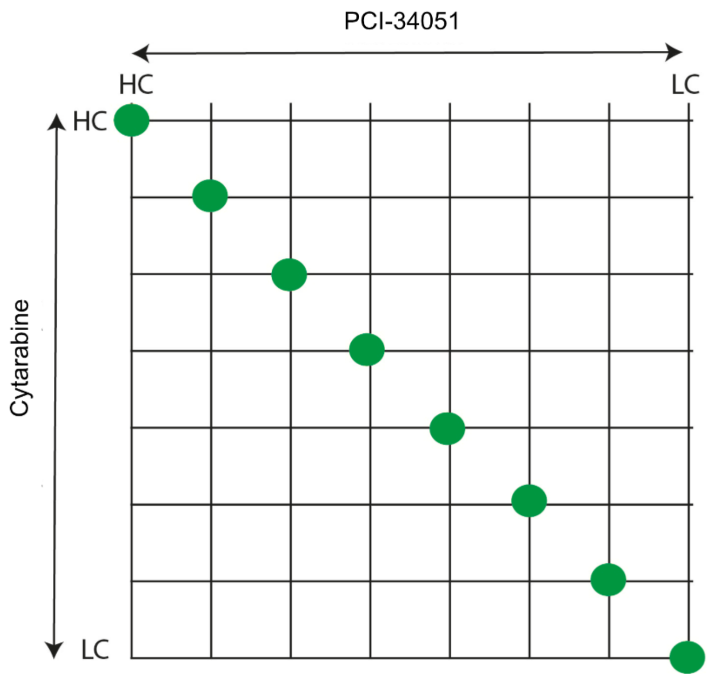

Figure S5: Combination treatment setting
Cells were exposed to the indicated drugs at decreasing concentration for 72h. Dots on the lines indicate data points used to determine the cytotoxicity effects. HC, highest concentration; LC, lowest concentration.

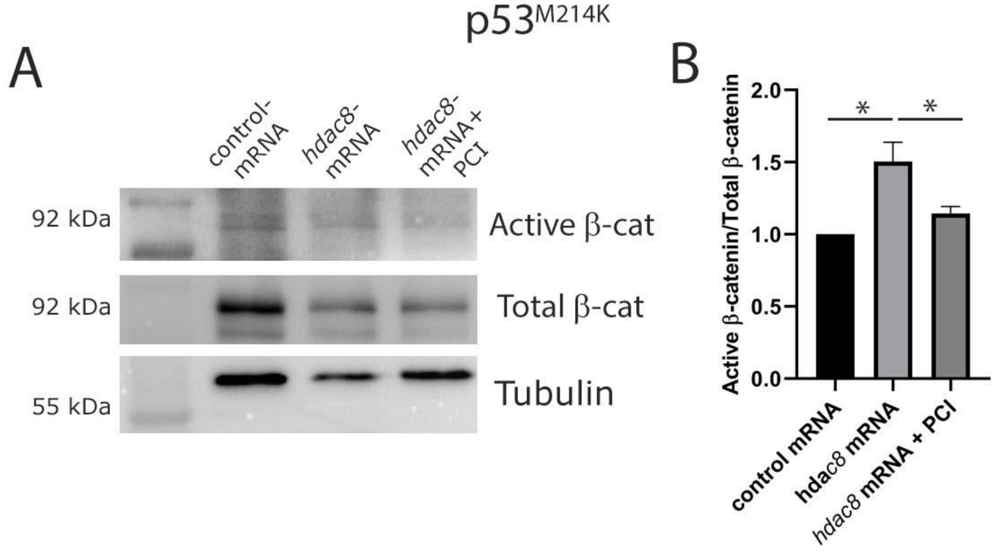

Figure S6: β-catenin activation in zebrafish p53^{M214K} embryos

(A-B) Western blot analyses of β-catenin activation status in control-mRNA, *hdac8*- and *hdac8*-injected PCI-treated embryos at 3 dpf. Histograms represent mean value ± SD from two independent experiments. *p<0.05, ANOVA One-Way followed by Tukey post-hoc correction.

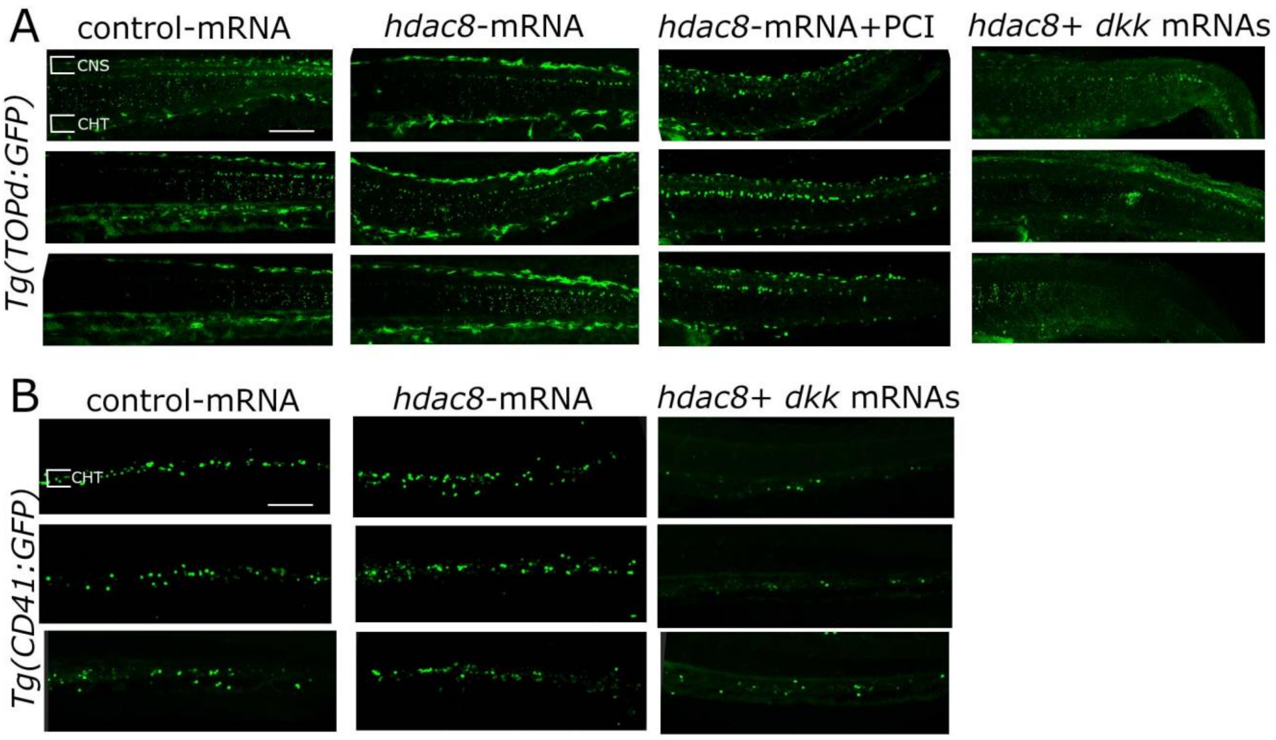

Figure S7: Canonical Wnt pathway modulation by HDAC8

(A) Representative images of canonical Wnt pathway modulation by *hdac8* overexpression, *dkk1b* injection and PCI treatment on HSPCs in the CHT of the Wnt reporter line *Tg(TOPdGFP)*. (B)

Representative images of canonical Wnt pathway modulation by *hdac8* overexpression and Wnt inhibition by PCI on HSPCs in the CHT of the reporter line *Tg(CD41:GFP)*. Scale bar represents 100 µm.

2.2 Supplementary tables

Supplementary Table 1

Primer list

**Primers human**	**Sequence 5'- 3'**
CCND1 FW	GAAGATCGTCGCCACCTG
CCND1 REV	GACCTCCTCCTCGCACTTCT
CMYC FW	CACCAGCAGCGACTCTGA
CMYC REV	GATCCAGACTCTGACCTTTTGC
GAPDH FW	CAACGACCACTTTGTCAAGC
GAPDH REV	CTGTGAGGAGGGGAGATTCA
NKD1 FW	TCGCCGGGATAGAAAACTACA
NKD1 REV	CAGTTCTGACTTCTGGGCCAC
PPP2R2B FW	CCTCATTCCGGCCAGGCTC
PPP2R2B REV	ATCCATGATCCCTCCCCGCA
**Primers zebrafish**	**Sequence 5'- 3'**
axin2 FW	GGCCACTGTAGTGGGTCTGT
axin2 REV	ATTAGGATTTCCGGGGTCAC
β-actin FW	CCGTGACATCAAGGAGAAG

β-actin REV	ATACCGCAAGATTCCATACC
baxa FW	CAACTGGGGAAGAGTTGTGG
baxa REV	ACCCTGGTTGAAATAGCCTTG
bbc3 FW	CCCACATCCCCTCACATGAT
bbc3 REV	TCTGTTCCTGAATTGTCCCTG
bida FW	CAGCGACCTACAGAGACCTT
bida REV	GCCTCTTCTGCATTGACTGA
cdkn1a FW	CCTGAGGAGATCTGAAACCC
cdkn1a REV	TGTGACAATATGTTTTGAGCTTCT
cmyb FW	GACACAAAGCTGCCCAGTTG
cmyb REV	GCTCTTCCGTCTTCCCACAA
gadd45ba FW	TGCATCCTCGTCACTAACTCT
gadd45ba REV	CAACGGCTCTCCTCACAGTA
gata2b FW	TCTGCTCGGAAACATGACGA
gata2b REV	ATTTACACATTCACGTCCCGAG
nkd1 FW	ACACATCCCGCTTTGGAACA
nkd1 REV	AACGGGTGGCGTGGGTAGGT
ppp2r2b FW	GTCTTCCAGAGAGAGCAGGAG
ppp2r2b REV	GCTCGTGGGTCTGGAAGGTCCT
spi1b FW	GCCATTTCATGGACCCAGG

spi1b REV	ACACCGATGTCCGGGGCAA
rpl8 FW	CTCCGTCTTCAAAGCCCATGT
rpl8 REV	TCCTTCACGATCCCCTTGATG

Supplementary Table 2

Antibodies for western blot analyses

**Primary antibody and catalogue number**	**Dilution**	**Company**
anti-total-SMC3 (rabbit)<br>SAB2701720	1:500	Sigma Aldrich, St. Louis, Missouri, US
anti-acetyl-SMC3 (mouse)<br>MABE1073	1:500	Merck-Millipore, Burlington, Massachusetts, US
anti-HDAC8 (rabbit)<br>sc-11405	1:500	Santa Cruz Biotechnology, Dallas, Texas, US
anti-active β-catenin (mouse), clone 8E7<br>05-665	1:500	Merck-Millipore
anti-total β-catenin (rabbit)<br>8480	1:1000	Cell Signaling Technology, Danvers, Massachusetts, US
anti-vinculin (mouse)<br>V9131	1:6000	Sigma-Aldrich
anti-tubulin (mouse)<br>T9026	1:2500	Merck-Millipore
Anti-RUNX1 (rabbit)<br>PB9157	1:1000	Boster Biological Technology, Pleasanton, California, US
**Secondary antibody**	**Dilution**	**Company**

HRP-conjugated goat anti-rabbit<br>7074	1:5000	Cell Signaling Technology
HRP-conjugated horse anti-mouse<br>7076	1:4000	Cell Signaling Technology
